# Assessment of nutritional status using anthropometric variables by multivariate analysis

**DOI:** 10.1186/s12889-019-7372-2

**Published:** 2019-08-05

**Authors:** Ankita Bhattacharya, Baidyanath Pal, Shankarashis Mukherjee, Subrata Kumar Roy

**Affiliations:** 1 Senior Research Fellow, Biological Anthropology Unit, Indian Statistical Institute, 203, B.T. Road, Kolkata, 700108 India; 2 Associate Scientist, Biological Anthropology Unit, Indian Statistical Institute, 203, B.T. Road, Kolkata, 700108 India; 3 Dept. of Physiology, University of Calcutta, 92 Acharya Prafulla Chandra Road, Kolkata, 700 009 India; 4 Professor, Biological Anthropology Unit, Indian Statistical Institute, 203, B.T. Road, Kolkata, 700108 India

**Keywords:** Nutritional status assessment, Anthropometric measurements, Confirmatory factor analysis, Discriminant function analysis

## Abstract

**Background:**

Undernutrition is a serious health problem and highly prevalent in developing countries. There is no as such confirmatory test to measure undernutrition. The objective of the present study is to determine a new Composite Score using anthropometric measurements. Composite Score was then compared with other methods like body mass index (BMI) and mid-upper arm circumference (MUAC) classification, to test the significance of the method.

**Methods:**

Anthropometric data were collected from 780 adult Oraon (Male = 387, Female = 393) labourers of Alipurduar district of West Bengal, India, following standard instruments, and protocols. Nutritional status of the study participants were assessed by conventional methods, BMI and MUAC. Confirmatory factor analysis was carried out to reduce 12 anthropometric variables into a single Composite Score (C) and classification of nutritional status was done on the basis of the score. Furthermore, all the methods (BMI, MUAC and C) were compared and discriminant function analysis was adopted to find out the percentage of correctly classified individuals by each of the three methods.

**Result:**

The frequency of undernutrition was 45.9% according to BMI category, 56.7% according to MUAC category and 51.8% according to newly computed Composite Score. Further analysis showed that Composite Score has a higher strength of correct classification (98.7%), compared to BMI (95.9%) and MUAC (96.2%).

**Conclusion:**

Therefore, anthropometric measurements can be used to identify nutritional status in the population more correctly by calculating Composite Score of the measurements and it is a non-invasive and relatively correct way of identification.

## Background

Human body needs a proper nutrition through well balanced diet to fulfill body requirements and to maintain basic body physiology. Improper nutrition leads to the consumption of excess calorie (over-nutrition) or insufficient supply of one or more essential nutrients (under-nutrition). Over-nutrition is a threat that increases body weight and causes several non-communicable diseases. On the other, undernutrition, caused due to the insufficient intake of energy and nutrients, is a serious health problem for the economically backward, developing countries like India [1]. It causes nutrition related complications, different deficiency diseases and even death by decreasing body immunity [2–5]. The short-term effect of undernutrition is weakness and recurring illness. Whereas, in the long run it hampers all vital functions causing low weight, growth retardation of children and adolescent, decreased immunity leading to recurring infections [6], occurrence of chronic diseases like diabetes mellitus, hypertension, and coronary heart diseases in later adult life [7] and impaired mental development [8]. Furthermore, in women, undernutrition may cause obstetric complications leading to maternal and infant mortality and increases the probability to give low birth weight babies and thus leading to the undernutrition cycle start again, spanning several generations [3, 9–11]. Besides, a chronic undernutrition also causes a reduced work capacity and ability to sustain economically productive work resulting in low income [12, 13]. Therefore, undernutrition is a critical burden and curse for the development of human being as well as for the society.

The main causes of undernutrition can be broadly classified as biological, behavioral and sociological factors [6, 3]. The biological causes may be infectious diseases like HIV/AIDS, TB etc. and also helminthes infestation which decreases intestinal nutrient absorption and thus developing poor nutrition. Behavioral factors include insufficient access to food, inadequate or inappropriate knowledge, practice and sanitation. The major social risk factors are political situation, lack of education and economic inequality [14–16]. Cultural influences on food habits along with several religious taboos and social customs may also cause nutritional deficiency [17–19].

Thus, undernutrition is a condition of poor nutritional status resulting from reduced food intake or impaired metabolism and evaluation of nutritional status is necessary to determine the severity of undernutrition. As there is no objective test to measure nutritional status, therefore numerous screening methods have been developed to determine the nutritional status of individual: (i) assessing clinical signs and symptoms, (ii) biochemical indicators (iii) dietary survey and (iv) anthropometric measurements. Assessment of clinical signs and symptoms need proper knowledge for evaluation, whereas biochemical indicators are relatively expensive and time consuming to perform in community level. On the other, dietary survey can give an idea of daily energy intake but there may have chances of misreporting and also need food consumption data of several days to obtain the estimate of usual diet.

Anthropometry has a long tradition of assessing nutritional and health status of adults as this is an inexpensive, non-invasive method that provides detailed information on different components of body structure, especially muscular and fat components [20, 21]. Moreover, anthropometric measurements are highly sensitive to the broad spectrum of nutritional status, whereas biochemical and clinical indicators are useful only at extremes of malnutrition. Among the widely used anthropometric measurements, body mass index (BMI) and mid-upper-arm-circumference (MUAC) are most significant and reliable.

BMI (Body mass index) is generally considered as a good indicator and used for the assessment of chronic energy deficiency of adults, especially in developing countries [22–24]. It is highly correlated with fat and fat-free mass and so the protein and fat reserves of body can be estimated. In normal adults the ratio is approximately constant, and a person with a low BMI is underweight for his/her height.

However, there are some difficulties associated with the sole use of BMI, for example the ratio of sitting height to standing height or cormic index can influence BMI [25]. Cormic index varies both between populations and within populations [26]. So, without the correction by cormic index as a correction factor, the sensitivity and specificity of BMI as an indicator of nutrition may be low. Age is another factor that may alter the functional significance of BMI at different ages; because adults tend to loose fat free mass and increase fat mass with increasing age [26–28]. Oedema can also affect the significance of BMI. Adults may develop oedema when severely undernourished, which artificially increases an individual’s weight resulting in BMI appearing more normal than the actual value [29]. Moreover, the universal cut-off of the BMI cannot be applicable across different populations [30]. So, these inabilities limit the usefulness of BMI as an accurate screening tool to assess adult undernutrition.

On the other, MUAC (Mid-upper arm circumference) is another important indicator for simple screening of adult nutritional status, specifically in developing countries [31, 32]. The measurement requires fewer apparatus and easy to perform even on the most debilitated individuals. It is independent of height and indicates the arm muscle and sub-cutaneous fat; both being important determinants of survival in starvation. Though classification of undernutrition according to the MUAC category is more appropriate than BMI category, but is not completely error free. Insufficient data are available correlating MUAC with undernutrition and other functional measures in adults, across different ethnic and population groups. Furthermore, the use of MUAC in adults may be affected by the redistribution of subcutaneous fat towards central areas of the body during aging [33]. Therefore, age-specific cut-off points of MUAC may be required. MUAC is also very sensitive towards intra- and inter- observer errors.

In view of the above, a more accurate and population specific method is necessary to assess the nutritional status of the population using anthropometric measurements. Present article documents the development of the method with specific statistical tools.

## Methods

### Study population

Anthropometric data were collected as part of an ongoing bio-medical project on Oraon labourers of Alipurduar district of West Bengal, India. Data includes 780 (Male 387, Female 393) adults from two occupational subgroups, one engaged in agriculture and other in tea garden, both having similar socio-economic status and living condition. The study was approved by the Ethical Committee of Indian Statistical Institute, Kolkata and was performed with the prior written consent from the participants. No statistical sampling was followed, because any kind of selection within the population would have raised suspicion in the minds of the people studied, regarding the purpose of the study. However, the participants were chosen without any conscious bias; actually the participants who could be persuaded to participate in the study and volunteered for participation in the study were included in the sample. Studies on the nutritional status of the indigenous population have an important significance in context of health planning. Thus, the present anthropometric measurements were collected from one of such indigenous group, Oraon in the Dooars foothill region of West Bengal.

### Anthropometric measurements

Anthropometric measurements were obtained following standard protocol and instrument [34]. These were two length measurements i.e. height (Ht.) and sitting height (SHt.), measured (0.1 cm) by Martin’s anthropometric rod (GPM, Switzerland). Weight (Wt.) was measured (0.1 kg) with an electronic scale (Omron HBF-375 Karada Scan, Japan). Five circumferences i.e. calf (CC), mid upper arm (MUAC), chest (CCN), waist (WC) and hip (HC) were measured by measuring tape. Five skinfolds i.e. calf (CSK), biceps (BSK), triceps (TSK), sub-scapular (SBSK) and supra-iliac (SISK) were measured (0.1 mm) on the left side of the body by Holtain skinfold caliper. Four diameters i.e. bi-epicondylar diameter of humerus (BDH), bi-condylar diameter of femur (BDF), bi-acromial diameter (BAD) and bi-iliac diameter (BID) were measured (0.1 cm) by sliding caliper and spreading caliper (GPM, Switzerland).

### Nutritional status

Nutritional status of the study population was assessed in terms of the two conventional methods i.e. BMI (Body mass index) [24] and MUAC (Mid-upper arm circumference) [32].

(BMI) has been calculated using the formula: $BMI=\frac{Weight\ (Kg.)}{Height^2\ \left({m}^2\right)}$

The criteria used for the classification of the nutritional status have been described in Table 1.

**Table 1:** Criteria for classification of nutritional status

Nutritional Category	BMI	MUAC (cm.)	
		Male	Female
Undernutrition	< 18.50	< 23.00	< 22.00
Normal	18.50–24.99	≥23.00	≥22.00
Obese	≥25.00	–	

As the mean of BMI and MUAC of the studied sample were 18.99 and 22.20 cm, so for the analytical purpose, nutritional status was classified into two categories; (a) Chronic energy deficiency or undernutrition and (b) normal.

### Statistical analysis

Initially, sixteen anthropometric measurements, which have previously been used for nutritional assessment, were used in the analyses. Among them twelve variables were found significant correlation with height, weight and MUAC. These were used for further statistical testing. Furthermore, by elimination method it was verified that the selected 12 variables can describe the highest variance of the newly computed variable. Thus these twelve variables created a single score variable that helped significantly for the assessment of nutritional status.

Descriptive statistics of all the variables were calculated. Then First Order Confirmatory Factor Analysis (CFA) and Discriminant Function Analysis were performed. All of the statistical analysis was carried out using PASW, version 18.0 (SPSS Inc., Chicago, IL, USA) and STATA, version 13.0 (STATA Corp, USA).

### Confirmatory factor analysis

Confirmatory factor analysis (CFA) helps to test the hypothesis that a relationship between observed variables and their underlying latent construct exists. This analysis is primarily a theory driven statistical data reduction technique used to explain covariance among different observed random variables and thereby reducing large number of variables to parsimonious and meaningful groups of underlying unobserved variables named factors [35]. The analysis gives a path diagram for the measurement model in STATA. In this study, there were twelve anthropometric variables (observed variables) in the rectangular boxes which have a commonality or shared variance or covariance. This covariance corresponds to the latent factor or latent variable. The observed anthropometric variables are related to the latent variable through factor loadings which are fundamentally regression coefficients. A part of variance of the indicators (observed variables) that cannot be explained by the latent factor is termed as measurement error of the model and therefore unique to each observed variable. The latent factor, observed variables and the measurement errors together describe a linear equation and can be expressed in matrix form as:

$$ X={\Lambda}_x\xi +\varepsilon, $$

Where X is a column vector of 12 standardized anthropometric variables, Λ_*x*_ is a 12 × 1 matrix of coefficients relating each variable to its latent factor, *ξ* is the latent variable and *ε* is the measurement errors, which is a 12 × 1 matrix.

$$ X=\left[\begin{array}{ccc}{X}_1& =& Ht.\\ {}{X}_2& =& Wt.\\ {}{X}_3& =& MUAC\\ {}{X}_4& =& CC\\ {}{X}_5& =& CC N\\ {}{X}_6& =& WC\\ {}{X}_7& =& HC\\ {}{X}_8& =& BSK\\ {}{X}_9& =& TSK\\ {}{X}_{10}& =& CSK\\ {}{X}_{11}& =& SBSK\\ {}{X}_{12}& =& SISK\end{array}\right],\kern0.5em {\Lambda}_x=\left[\begin{array}{c}{\lambda}_1\\ {}{\lambda}_2\\ {}{\lambda}_3\\ {}{\lambda}_4\\ {}{\lambda}_5\\ {}{\lambda}_6\\ {}{\lambda}_7\\ {}{\lambda}_8\\ {}{\lambda}_9\\ {}{\lambda}_{10}\\ {}{\lambda}_{11}\\ {}{\lambda}_{12}\end{array}\right],\in =\left[\begin{array}{c}{\in}_1\\ {}{\in}_2\\ {}{\in}_3\\ {}{\in}_4\\ {}{\in}_5\\ {}{\in}_6\\ {}{\in}_7\\ {}{\in}_8\\ {}{\in}_9\\ {}{\in}_{10}\\ {}{\in}_{11}\\ {}{\in}_{12}\end{array}\right],\kern0.5em $$

Thus latent variable was calculated from each linear equation for each individual and was termed as Composite Score. Later Composite Score was used as an alternative variable instead of the 12 anthropometric variables (observed variables) and a new classification was developed on the basis of this score. Negative Composite Scores were considered as undernutrition and positive Scores as normal nutritional status.

### Discriminant function analysis

Discriminant function analysis is primarily a multivariate test to observe the differences between groups. This is the reverse of MANOVA, where the independent variables are the continuous predictors and dependent variables are the groups [36].

The analysis can be split into 2-steps- (a) testing significance of a set of discriminant functions, and, (b) classification. In the study, second step of the analysis was used for the classification of nutritional status in view of the equations created in the analysis. Computationally a canonical correlation analysis was performed and that determined the successive functions and canonical roots. Classification was then possible from the canonical functions. Individuals were classified in the groups in which they had the highest classification scores [37]. This analysis further provided a percentage of overall correct classification.

## Result

Table 2 depicts the descriptive statistics of age, selected anthropometric traits and body mass index of the study population of either sex. It was observed that except the mean skinfold values, all other mean values were higher in males than females which indicate the poor nutritional status of women labourers of the present study group.

**Table 2:** Descriptive statistics of age, BMI and selected anthropometric traits in either sex of adult individuals

Variables	Male (*n* = 387)		Female (*n* = 393)		Total (*n* = 780)	
	Mean	S.D.	Mean	S.D.	Mean	S.D.
Age (yr.)	35.85	14.14	35.99	13.75	35.92	13.94
Height (Ht.) (cm.)	162.05	5.26	150.50	5.17	156.23	7.78
Weight (Wt.) (kg.)	50.78	7.18	42.34	7.60	46.53	8.51
Mid upper arm circumference (MUAC) (cm.)	23.18	3.18	21.38	4.61	22.20	3.58
Calf Circumference (CC) (cm.)	30.02	4.21	27.42	4.13	28.71	4.37
Chest Circumference, normal (CCN) (cm.)	81.45	7.25	71.04	6.80	76.20	8.74
Waist Circumference (WC) (cm.)	71.15	6.78	70.18	9.26	70.66	8.14
Hip Circumference (HC) (cm.)	80.66	7.56	80.14	7.75	80.40	7.65
Biceps Skinfold (BSK) (mm.)	3.03	1.83	4.23	3.37	3.63	2.78
Triceps Skinfold (TSK) (mm.)	5.28	2.43	8.37	4.36	6.84	3.85
Calf Skinfold (CSK) (mm.)	6.15	3.86	8.99	4.51	7.58	4.43
Sub scapular Skinfold (SBSK) (mm.)	7.68	2.90	9.81	5.12	8.75	4.30
Supra iliac Skinfold (SISK) (mm.)	3.76	1.71	5.80	7.36	4.79	5.46
Body Mass Index (BMI)	19.33	2.49	18.66	3.09	18.99	2.83

**Fig. 1:**
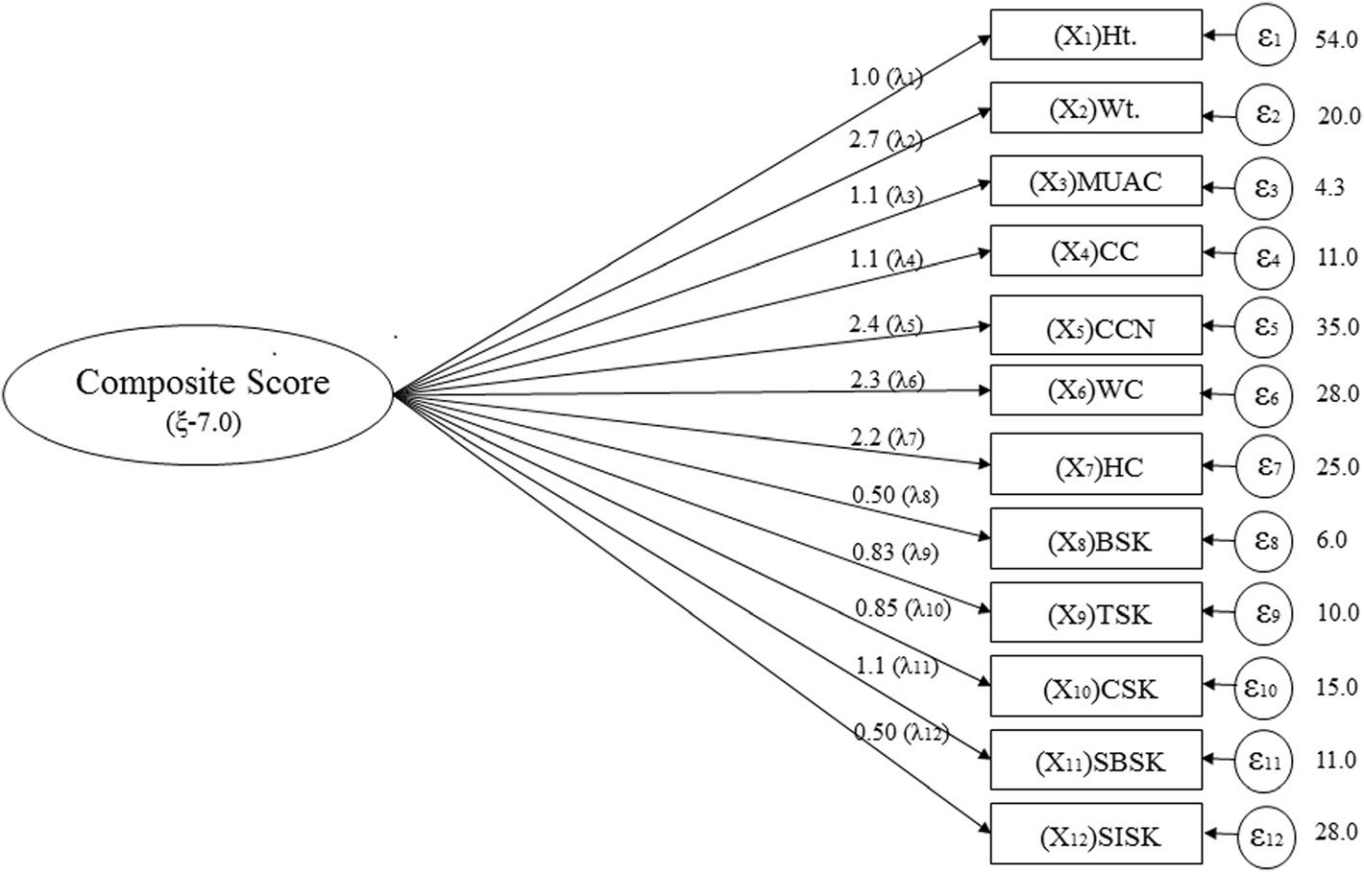
Path diagram of the Measurement Model of Confirmatory factor analysis. Describes the path diagram of measurement model of the confirmatory factor analysis. The variance of Composite Score (ξ) was 7.0. The values of the respective factor loadings (λ_i_) were mentioned and the measurement error (ɛ_i_) of the model for each respective observed variable was also calculated as shown

Table 3 is the tabular form of the path diagram (Fig. 1), it depicts the values of coefficients and measurement errors of the model, which were used to compute the Composite Score in the analysis.

**Table 3:** Factor loadings of Observed variables in Confirmatory factor analysis

Variables (X)	Coefficients (λ_i_)	Measurement error term (ɛ_i_)
Height (Ht.)	1.0	54.0
Weight (Wt.)	2.7	20.0
Mid upper arm circumference (MUAC)	1.1	4.3
Calf Circumference (CC)	1.1	11.0
Chest Circumference, normal (CCN)	2.4	35.0
Waist Circumference (WC)	2.3	28.0
Hip Circumference (HC)	2.2	25.0
Biceps Skinfold (BSK)	0.50	6.0
Triceps Skinfold (TSK)	0.83	10.0
Calf Skinfold (CSK)	0.85	15.0
Sub scapular Skinfold (SBSK)	1.1	11.0
Supra iliac Skinfold (SISK)	0.5	28.0

Table 4 depicts the frequencies and percentages of individuals categorized under undernutrition and normal category considering BMI, MUAC and Composite Score of the present analyses. It was observed that according to BMI 40.1% males and 51.7% females were categorized as undernutrition. Whereas the percentage (Male = 42.9% & Female = 70.2%) of undernutrition increased using MUAC scale. However, Composite Score classified 38.2% males and 65.1% females under undernutrition. The differences of frequencies (undernutrition and normal) classified by BMI and Composite Score were found significant (z-test for equality of proportion was done) only for females.

**Table 4:** Classification of Nutritional status according to BMI, MUAC and Composite Score

Variable	Undernutrition									Normal								
	Male (*n* = 387)		*p* ^*^	Female (*n* = 393)		*p* ^*^	Total (*n* = 780)		*p* ^*^	Male (*n* = 387)		*p* ^*^	Female (*n* = 393)		*p* ^*^	Total (*n* = 780)		*p* ^*^
	No.	%		No.	%		No.	%		No.	%		No.	%		No.	%	
BMI	155	40.1	0.603	203	51.7	***< 0.01***	358	45.9	***0.019***	232	59.9	0.603	190	48.3	***< 0.01***	422	54.1	***0.019***
Composite Score	148	38.2		256	65.1		404	51.8		239	61.8		137	34.9		376	48.2	
			0.187			0.126			0.054			0.187			0.126			0.054
MUAC	166	42.9		276	70.2		442	56.7		221	57.1		117	29.8		338	43.3	

*z-test for equality of proportions was carried out

Table 5 gives the values of coefficients of discriminant function analysis and also assesses how well the Fisher’s classification function coefficients were classified between the groups. The coefficients were used to construct a discriminant function for each group, i.e. under nutrition and normal.

**Table 5:** Fisher’s classification function coefficients of Discriminant Function Analysis to predict nutritional status

Classification Categories		Classification	
		Undernutrition	Normal
BMI	Coefficient	4.802	6.030
	Constant	−40.753	−63.862
MUAC	Coefficient	3.660	4.672
	Constant	−36.970	− 59.798
Composite Score	Coefficient	−0.741	0.799
	Constant	−1.395	−1.507

For Composite Score

1 $$ \mathrm{Undernutrition}=-1.395+\left(-0.741\right)\ast Composite\ Score $$

2 $$ \mathrm{Normal}=-1.507+0.799\ast Composite\ Score $$

Equations (1) and (2) have to be calculated for each case to get the undernutrition or normal value and the case will be classified for which computed value will be higher. For example, if eq. (2) gives the higher value for a particular case, then the case will be classified as normal.

Table 6 describes the result of discriminant factor analysis. It was observed that according to the BMI classification, 7.6% were misclassified as normal. According to MUAC category, 6.8% was misclassified as undernutrition. For Composite Score misclassification were only 2.7, who were wrongly classified as normal. Furthermore, it was observed that according to the BMI scale the overall correct prediction was 95.9%, and by MUAC scale it was 96.2%. In comparison with BMI and MUAC classification, the newly computed Composite Score has the higher strength (98.7%) of classifying overall nutritional status.

**Table 6:** Classification results of nutritional status with three independent methods

BMI Categories			*Predicted*		Total	Overall correctly classified
			Under nutrition	Normal		
*Observed*	Count	Undernutrition	358	0	358	95.9%
		Normal	32	390	422	
	%	Undernutrition	100	0	100.0	
		Normal	7.6	92.4	100.0	
MUAC Categories						
*Observed*	Count	Under Nutrition	412	30	442	96.2%
		Normal	0	338	338	
	%	Under Nutrition	93.2	6.8	100.0	
		Normal	0	100.0	100.0	
Composite Score						
*Observed*	Count	Under Nutrition	405	0	405	98.7%
		Normal	10	366	376	
	%	Under Nutrition	100.0	0	100.0	
		Normal	2.7	97.3	100.0	

## Discussion

The present study tries to find out the most precise way of assessing nutritional status. Anthropometry has long been used as indicator of nutritional status because it is non-invasive and less expensive. Anthropometric measurements help in calculating both BMI and MUAC, which provide a simple and convenient value for assessing nutritional status. Nutritional assessments in rural population usually rely on BMI and MUAC, as it does not require much instruments, time and efficiency; but have independent limitations. For example, individuals with ectomorphic somatotype may be misclassified as undernourished with BMI classification. Again, human body has bilateral asymmetry; therefore, taking MUAC measurement on one side may provide erroneous assessment. On the other, variation of MUAC is very high depending on the physical activity and food intake of the individual. Moreover, both BMI and MUAC do not have population specific cut-off values. Therefore, there are every chances of misclassifying nutritional status of individuals.

The effort of the present study was to develop a better and rigorous tool that can easily identify nutritional status. A number of anthropometric measurements were used to construct a new method for assessing nutritional status. All the anthropometric traits were tested for its association to height, weight and MUAC, however, found 12 anthropometric traits that have precise and good association with height, weight and MUAC. Those 12 anthropometric traits represented into one Composite Score by confirmatory factor analysis (Fig. 1). Later, classification was done on the basis of Composite Score and was compared with other two classification categories (BMI and MUAC) by discriminant function analysis to find out which one gives the best classification.

A separate equation for undernutrition and normal was obtained from discriminant function analysis (Table 5) for BMI, MUAC and Composite Score which was used to predict the respective frequencies. In comparison with BMI and MUAC category, it was observed (Table 4) that frequency of undernutrition and normal (well-nourished) individuals significantly vary when categorized with Composite Score and the difference in frequency was found higher in case of females than males. It may be because the visceral redistribution of fat predominantly affects females [38] thereby causing differences in fat patterning between sexes [39]. Therefore disparity in measurements is always prominent in females. Then the same analysis was also used to test the strength of the classification category on the basis of observed and predicted values (Table 6). It was observed that the newly computed Composite Score qualifies for the highest strength (98.7%) to identify the individuals in the specific category followed by MUAC (96.2%) and BMI (95.9%). This may be because; as the newly computed Composite Score is a multidimensional method of nutritional assessment therefore it may have maximum chances of correct classification.

The assessment of nutritional status by anthropometry was previously done primarily on the basis of BMI or MUAC [31, 40, 20, 41]. Some studies also used skinfold measurement like BSK, TSK and CSK [42, 43, 32, 44] for the nutritional assessment. Waist circumference and waist-hip ratio was also used for nutritional assessment [45, 46]. But there is hardly any study that have used the linear measurements like Ht., circumferences like MUAC, CC, CCN, WC and HC, skinfolds like BSK, TSK, CSK, SBSK, SISK and Wt. to predict nutritional status or have been tried to group those. To our knowledge, it is hard to find comparable literatures on this issue because it is a new one.

Moreover, the cut-offs of all the nutritional assessment tools with anthropometric traits have same values, irrespective of ethnicity. Adult individuals of different ethnic backgrounds display differences in body shape and cormic index. Studies found a higher percentage of body fat at lower BMI in case of Asians [47]. So, it may be inappropriate to compare different population with a single universal reference value.

But in calculating the Composite Score, the values of anthropometric data are being used of the study population, so the classification category is more specific for that study group. Finally, it is believed that the newly developed method will be useful in identifying nutritional status of the individuals of a particular study group more correctly.

### Limitations

The present study tried to assess the nutritional status of a particular ethnic group with selected anthropometric measurements by computing Composite Score. There are few limitations also in calculating such Composite Score; it needs some statistical computation which may not always be feasible in the field situation. Moreover, the method is applicable to identify only the under-nourished and normal individuals, not the obese individuals. In the present data, as there were no obese individuals, so this method has been applied successfully. Further study in different population with more sample size is needed to classify the nutritional status for three categories following the present Composite Score method.

## Conclusion

The adverse effect of undernutrition on health, development and economic productivity is well established. It needs proper and accurate identification to get the idea of severity to address the issue. The newly computed Composite Score can predict the nutritional status more accurately than BMI and MUAC. Moreover, it will give population based cut-off values which will lower the probability of mis-classification. Lastly, it is expected that scientific ventures will continue to develop such scores with the data of other population groups and it will provide a comprehensive understanding over this newly developed method.

## Abbreviations

BAD: Bi-acromial diameter
BDF: Bi-condylar diameter of femur
BDH: Bi-epicondylar diameter of humerus
BID: Bi-iliac diameter
BMI: Body mass index
BSK: Biceps skinfold
CC: Calf circumference
CCN: Chest circumference (Normal)
CFA: Confirmatory factor analysis
CSK: Calf skinfold
HC: Hip circumference
Ht: Height
MUAC: Mid upper arm circumference
SBSK: Sub-scapular skinfold
SHt: Sitting height
SISK: Supra-iliac skinfold
TSK: Triceps skinfold
WC: Waist circumference
Wt: Weight
